# The Moderating Effect of Price on the Relationship Between Environmental Attitude and the Purchase Behavior of Organic Products

**DOI:** 10.3390/foods14203550

**Published:** 2025-10-18

**Authors:** Iris Castillo-Plaza, Nelson Carrión-Bósquez, Andrés García-Umaña, Oscar Ortiz-Regalado, Ximena Tobar-Cazares, Franklin Naranjo-Armijo, Cristina Villacís-Mejía, Lenin Tobar-Cazares, Rodrigo Arévalo-Mejía

**Affiliations:** 1 Carrera de Mercadotecnia, Facultad de Ciencias Empresariales, Universidad Técnica Estatal de Quevedo, Quevedo 120550, Ecuador; icastillop@uteq.edu.ec; 2 Departamento de Administración, Facultad de Economía y Administración, Universidad Católica del Norte, Antofagasta 1270709, Chile; 3 Facultad de Administración y Economía, Escuela de Diseño e Innovación Tecnológica, Universidad de Tarapacá, Arica 1000007, Chile; agarciau@academicos.uta.cl; 4 Escuela Profesional de Ingeniería en Agronegocios, Universidad Nacional de Cajamarca, Cajamarca 06001, Peru; oortizr@unc.edu.pe; 5 Facultad de Derecho, Ciencias Administrativas y Sociales, Universidad UTE, Quito 170508, Ecuador; ximena.tobar@ute.edu.ec (X.T.-C.); rodrigo.arevalo@ute.edu.ec (R.A.-M.); 6 Vicerrectorado Administrativo Financiero, Vicerrectorado Académico, Universidad Pública de Santo Domingo de los Tsáchilas—UPSDT, km 28, vía Santo Domingo—Quevedo, Santo Domingo 230153, Ecuador; fnaranjoarmijo@upsdt.edu.ec (F.N.-A.); mvillacismejia@upsdt.edu.ec (C.V.-M.); 7 Facultad de Ciencias Económicas, Universidad Central del Ecuador, Quito 170515, Ecuador; ljtobar@uce.edu.ec

**Keywords:** SOR framework, environmental attitude, price, purchase behavior, organic products

## Abstract

Although previous research has highlighted the role of environmental concerns, responsibility, knowledge, and social norms in shaping sustainable consumption, the persistence of the intention–action gap remains a key challenge, particularly in Latin America. In this context, this study analyzes the moderating effect of price on the relationship between environmental attitude and its influence on the purchase behavior of organic products in an emerging market setting. To address this issue, a quantitative, cross-sectional survey was conducted with 329 Ecuadorian consumers of organic products, using validated scales adapted from prior studies. The proposed model was tested using partial least squares structural equation Modeling. The results confirm that environmental concern, environmental responsibility, subjective norms, and environmental knowledge positively influence environmental attitudes and that environmental attitude directly affects the purchase behavior of organic products. Importantly, price emerged as a critical moderator, showing that, even when consumers hold favorable environmental attitudes, higher prices significantly constrain their translation into purchasing behavior. This study adds theoretical originality by extending the relationship between environmental attitude and purchase behavior of organic products through the SOR framework and by evidencing in an emerging market context; while EA foster organic purchasing, this relationship is conditioned by the price, a situational factor often overlooked in research from developed economies.

## 1. Introduction

In recent decades, environmental degradation and growing concerns regarding pollution have transformed consumption patterns across multiple markets [1]. In this context, the search for responsible consumption alternatives has fueled interest in organic products, whose production is associated with environmentally friendly practices and health benefits [2]. However, although Environmental Attitudes (EA) have steadily increased, a persistent gap remains between what consumers declare in terms of intention and what they actually carry out in their purchasing decisions [3]. This mismatch underscores the need to understand the determinants of EA and its impact on the Purchase Behavior of organic products (PBOP), while also considering the role of external factors that may shape this relationship.

In Latin America, particularly in emerging markets such as Ecuador, the acquisition of organic products has shown steady growth; however, it remains a niche compared to the mass consumption of conventional foods [4]. This scenario reveals a contradiction: while consumers express greater concern about the climate crisis, environmental degradation, and the need for responsible consumption, sales figures do not always reflect this interest [5]. Thus, a tension arises between individuals’ willingness to act according to environmental values and the practical barriers they face when making purchasing decisions, with Price (PR) being one of the most significant [6,7]. This context underscores the importance of examining the factors that shape EA and its translation into PI within an environment in which sustainability is becoming a priority for both public policies and business strategies.

EA can be defined as a favorable or unfavorable psychological predisposition toward behaviors that promote environmental protection and preservation [8,9,10]. In the consumption domain, this attitude is closely linked to the purchase intention for sustainable products, understood as the likelihood that an individual will acquire a good in the future as a result of their beliefs, emotions, and social norms [6,11]. Accordingly, when consumers hold positive EA, they tend to place greater value on organic products, perceiving their choice as a contribution to mitigating the ecological impact and fostering a responsible lifestyle [12,13,14,15]. Nevertheless, purchase intention, although a strong predictor of actual behavior, is subject to situational constraints that may prevent its realization [3,16].

The main determinants of EA are Environmental Concern (EC), Environmental Responsibility (ER), Subjective Norms (SN), and Environmental Knowledge (EK) [17,18,19]. EC refers to the extent to which individuals recognize ecological problems and the need to adopt sustainable behaviors [20,21]. ER alludes to the personal perception of a moral obligation to care for the environment [22,23]. SN, in turn, is related to perceived social influence or the pressure exerted by family, friends, or reference groups that either encourage or discourage responsible consumption [6,7]. Finally, EK involves the level of understanding of ecological processes, consequences of consumption practices, and benefits of choosing sustainable products [3,24]. Together, these variables shape EA, and consequently, purchase intentions.

According to the ProEcuador portal, 58% of consumers purchase natural and organic products because they consider them healthier and contain fewer chemicals, which is even stronger among younger generations, as 89% of Generation Z and 85% of millennials reported having purchased them in the past six months. During this same period, there was a 23% increase in natural products and a 22% increase in organic products [25]. Despite consumers’ interest in adopting behaviors that minimize environmental impacts, the PR of organic products is a restrictive factor that hinders the transition from intention to action [3,19,26,27]. PR is understood as the monetary amount a consumer must sacrifice to obtain a good or service, and it plays a central role in purchase decisions, particularly in contexts where purchasing power is limited [6,7]. Thus, even when a favorable attitude toward organic products exists, their higher PR compared to conventional alternatives constitute a significant barrier that prevents their mass consumption [28,29]. This dilemma reveals the tension between the EA and the economic constraints in the PBOP.

Ecuador provides a particularly relevant context for analyzing EA and PBOP in emerging markets. Culturally, Ecuador represents a society in transition between traditional consumption patterns and increasing environmental awareness, driven by the diffusion of sustainability discourses through education, social media, and governmental initiatives [3,4]. Economically, the country exhibits structural characteristics typical of emerging economies, such as income inequality, sensitivity PR, and limited availability of certified organic products [6] factors that directly influence the translation of EA into purchasing behavior [7]. Moreover, Ecuador has implemented a National Strategy for Sustainable Production and Consumption aligned with the UN Sustainable Development Goals (SDG 12), creating an institutional framework that encourages responsible consumption while confronting barriers related to affordability and market development. Thus, studying Ecuador not only contributes empirical evidence from a Latin American perspective, which remains underrepresented in the literature, but also enables the examination of how cultural and economic conditions mediate the attitude–behavior gap in sustainable consumption.

To refine the identification of the research gap, the study emphasizes that most empirical evidence on green consumption based on the SOR model originates from developed economies and does not explicitly model PR as a moderator of the EA and PBOP. Moreover, the literature review evidences that previous studies rarely compare the relative contributions of EC, ER, SN, and EK under real affordability constraints. In Latin America, and particularly in Ecuador, there coexist underexplored structural conditions: high sensitivity PR in food consumption, limited dissemination and heterogeneous trust in organic certifications, fragmented supply and intermittent availability, and informality in sales channels; all of which intensify the attitude–behavior gap [3,6,7,8,9].

In view of the above, the research problem centers on demonstrating that, despite the presence of factors shaping consumers’ EA, purchasing behavior is conditioned by PR. Therefore, the objective of this study was to analyze the moderating effect of PR on the relationship between EA and PBOP. Within this context, the justification of this study lies in providing empirical evidence that can enable companies, policymakers, and social organizations to design more effective strategies to promote sustainable consumption, overcome economic barriers, and strengthen the transition toward environmentally responsible markets. To achieve this research objective, this study addresses the following sub-questions: (a) How do EC, ER, SN, and EK influence consumers’ EA? (b) How does EA influence the PBOP? (c) How does PR moderate the relationship between EA and PBOP?

## 2. Literature Review

Several theoretical frameworks have been developed to explain consumer behavior and the formation of EA, including the Theory of Planned Behavior, the Norm Activation Model, and the Value–Belief–Norm Theory [3,6,7]. Although these models highlight cognitive and normative processes, they often neglect the influence of external factors, such as EC, ER, SN, EK, and PR. In this regard, the Stimulus Organism Response (SOR) model expands the analysis by integrating external and internal environmental stimuli. Thus, this study adopts the SOR framework to examine how EC, ER, SN, and EK (stimuli) influence EA (organism), which in turn affects PBOP (response). Finally, it also tested how PR (stimulus) conditions the relationship between EA (organism) and PBOP (response).

### 2.1. Stimulus–Organism–Response Model

The SOR paradigm has been widely used to analyze human reactions to various stimuli [30,31,32]. Within the sphere of environment-related consumption, this model provides a broader understanding of how environmental factors shape consumer perceptions, and in turn, generate specific responses [33]. Despite its extensive use in traditional marketing contexts, its application to environmental issues and the adoption of green products remains relatively scarce [34]. In this framework, the stimulus represents external triggers such as messages, environments, or interactions that provoke reactions in individuals [35]. The organism refers to internal cognitive and affective processes, including perceptions, beliefs, and attitudes [31,32], whereas the response corresponds to observable behaviors, such as purchase decisions [36]. Within sustainable marketing, this approach is particularly useful in explaining how extrinsic and intrinsic factors foster EA that eventually guide the purchase of organic products [6,17].

The study employs the SOR model to demonstrate that PR, in addition to being a restrictive element, functions as a contextual stimulus that shapes the relationship between the organism and the response. This conceptual clarification establishes that affordability cues belong to the stimulus stage and moderate EA–PBOP relation, thus specifying the mechanism through which the gap between intention and action persists among organic product consumers. In light of this, the present study addresses the SOR framework from three perspectives. First, it jointly models EC, ER, SN, and EK as concurrent stimuli and estimates their relative contributions to EA, clarifying their comparative relevance under affordability constraints. Second, it theorizes and empirically tests PR as a contextual stimulus moderating the EA–PBOP relationship, thereby identifying the precise role of PR within the SOR chain. Third, it validates this structure in an emerging Latin American market, demonstrating that despite consumers’ favorable attitudes toward environmentally aligned consumption, PR becomes a conditioning factor that prevents consumers from abandoning traditional consumption habits, relegating the purchase of organic products to a secondary priority.

### 2.2. Environmental Concern

EC is recognized as one of the most relevant cognitive constructs for predicting purchasing behaviors aligned with environmental protection [37]. This concept encompasses both public and private awareness of the environment, which drives individuals to adopt responsible behaviors [38]. From this perspective, EC translates into the avoidance of products or services that may cause significant harm to the environment, and the development of positive attitudes toward the consumption of products considered organic [39,40,41].

Several studies have highlighted the role of EC as a key antecedent of green purchasing behavior. For instance, prior research has confirmed that EC is positively associated with purchase intention for organic products [42,43] as well as with the adoption of responsible consumption practices [44]. Similarly, Khan and Mohsin [45] found that environmental values, consumer interests, and social values strengthen the positive relationship between EC and pro-environmental behaviors. Moreover, the literature shows that a high level of EC can shape beliefs and behavioral actions by revealing that the environment is at risk and that changes in consumption habits are necessary [46]. In this sense, EC emerges as an indispensable construct for understanding how EA translates into concrete green purchasing decisions, thus justifying its inclusion as a central variable in theoretical models seeking to explain sustainable consumption behavior. To expand the empirical findings and contribute to the understanding of consumer behavior toward organic products, the following hypotheses were tested:

**Hypothesis** **1** **(H1).** *Environmental Concern influences consumers’ Environmental Attitude.*

### 2.3. Environmental Responsibility

ER is understood as a sense of duty and commitment toward protecting the environment [47]. This construct reflects individuals’ willingness to engage in responsible behaviors that contribute to improving environmental quality and ensuring the conservation of natural resources [48]. In this regard, those with higher levels of ER demonstrate a greater disposition to participate in pro-environmental behaviors, thereby fostering ecological protection and strengthening the link between individual responsibility and concrete actions aimed at safeguarding the environment [49,50,51,52,53].

Several studies have shown that ER has a significant impact on green purchase intention and behavior. For instance, Lee [54] demonstrated in Hong Kong that ER notably influences adolescents’ green purchasing activity, confirming that this construct is relevant even in the early stages of life. Likewise, research has documented that the intention to assume individual responsibility is reflected in consumers’ willingness to pay higher PR for sustainable products [55,56]. Furthermore, it has been noted that women tend to display a stronger inclination than men to actively engage in solving environmental problems, which underscores the importance of analyzing ER from socio-demographic perspectives [57]. Taken together, empirical evidence supports the notion that ER not only predicts green purchasing behavior [15], but also promotes a culture of social environmentalism in which consumers and organized groups assume conservation as a shared duty [58,59,60]. In light of the above and with the aim of providing further empirical evidence on this variable, the study sought to test the following hypothesis:

**Hypothesis** **2** **(H2).** *Environmental Responsibility influences consumers’ Environmental Attitude.*

### 2.4. Subjective Norms

SN is understood as the social influence exerted by society on the development of a specific behavior [3,61,62]. This construct consists of two dimensions: normative belief, which reflects the individual’s perception of how others expect them to act in a given situation, and motivation to comply, which refers to the desire to conform to social expectations [63,64,65,66]. Empirical literature has shown that SN exerts a significant influence on various pro-environmental behaviors, including the purchase intention for organic products [3,12,42,63,64,66,67,68]. Recent studies indicate that young consumers, particularly millennials, tend to guide their green consumption decisions based on perceived social expectations [3,7]. However, the findings remain inconclusive: while research such as that of Wang et al. [68] confirms a positive effect of SN on green purchasing, other authors have questioned their predictive role. Thøgersen and Zhou [69], Paul et al. [70], and Kumar et al. [71] argue that SN does not have a significant effect on the purchase intention of organic products, such as organic foods. Similarly, Taufique and Vaithianathan [72] concluded that the influence of SN is insignificant in shaping behavioral intention. These contradictory results reveal that, although SN represents a form of social pressure toward sustainable consumption, there is still no consensus regarding their true capacity to shape attitudes and consumption behaviors aligned with environmental protection [73,74]. To provide empirical evidence that may help resolve these contradictions, this study tested the following hypotheses:

**Hypothesis** **3** **(H3).** *Subjective Norms influences consumers’ Environmental Attitude.*

### 2.5. Environmental Knowledge

EK refers to an individual’s awareness, understanding, and perception of environmental problems and ecosystems, as well as the practical skills to address them [75,76]. This knowledge not only enables the identification of ecological effects derived from the production and use of goods [77], but also incorporates values and associations linked to sustainability and the protection of key resources [78,79,80]. Empirical evidence has consistently identified EK as a relevant antecedent of EA and green purchasing behavior. Previous studies have revealed that higher levels of EK are positively associated with EA [79,81,82,83] and with the purchase of sustainable products, reinforcing its role as a key determinant of green consumption [76]. Moreover, it has been observed that consumers with high EK levels have stronger perceptions of green marketing and eco-sustainable products, which drives their preference for responsible options [44,84]. EK and EA have been consolidated as the most influential predictors of green purchasing behavior, with EA acting as a mediating variable between EK and green purchasing [85]. Research conducted in various contexts, such as the Asian market, shows that increases in EK contribute to improving environmental performance and the willingness to purchase ecological products [86,87]. Therefore, EK not only precedes EA but also enhances individuals’ capacity to monitor their own purchasing behavior, thereby strengthening the transition toward more sustainable consumption patterns [71,88,89]. To provide further empirical evidence on the impact of this variable in the context of organic product consumption, the following hypothesis was tested:

**Hypothesis** **4** **(H4).** *Environmental Knowledge influences consumers’ Environmental Attitude.*

### 2.6. Environmental Attitude

EA is defined as an individual’s mental and emotional disposition toward the environment, manifested through their perceptions, evaluations, and concrete actions [7]. This construct constitutes an essential intrinsic factor in shaping pro-environmental behaviors and preferences for organic products, as it reflects not only ecological concerns but also the motivation to act consistently with such values [7,12,62,72]. The empirical literature has demonstrated that EA is a significant determinant of organic purchasing behavior across various contexts. Aertsens et al. [90] found a positive correlation between consumers’ attitudes and their consumption of organic food, while Von Meyer et al. [91] and Prakash et al. [92] confirmed this direct relationship among German consumers. Similarly, Kumar et al. [71] suggested that a favorable attitude toward organic products mediates the effect of EK on purchase decisions, thereby underscoring its integrative role within the decision-making process. Bon and Byoungho [93], in a comparative study between the United States and China, concluded that attitudes significantly influenced organic purchasing. Furthermore, recent research has reinforced that a positive pro-environmental attitude increases green purchase intention, especially among millennials who display a stronger predisposition to opt for sustainable alternatives when their attitude toward the environment is strong and consistent [3,6,7,8,9,12,94,95,96]. To further expand the empirical evidence in this field, this study sought to test the following hypotheses:

**Hypothesis** **5** **(H5).** *Environmental Attitude influences Purchasing Behavior of organic products.*

### 2.7. Price

PR constitutes one of the most decisive variables in shaping green purchase intention and behavior, as it represents both a perception of value and a potential barrier to the acquisition of sustainable products [7,26]. Although consumers may express environmental concerns, purchasing decisions are often conditioned by the perception that ecological or organic products are more expensive than conventional alternatives [6,67,97,98,99,100]. The empirical evidence reveals an ambivalent position regarding the role of PR in green consumption. On one hand, studies such as Naderi and Van [101] show that a significant proportion of millennials are willing to pay more for environmentally responsible products, highlighting the power of EA in overcoming economic barriers. On the other hand, other studies argue that high PR remains one of the main restrictive factors in the purchase of ecological products [19,67,98]. It has even been noted that the perception of economic inaccessibility may weaken green purchase intention, particularly among consumers who consider that sustainable products do not justify their value compared with conventional ones [102,103]. This divergence in findings underscores the need to deepen the analysis of PR as a critical variable, and specifically to identify that although consumers hold Environmental Attitudes, they may be overshadowed, thereby affecting their purchasing behavior for organic products due to the high PR of these goods. Therefore, this study tested the following hypotheses:

**Hypothesis** **6** **(H6).** *Price moderates the relationship between environmental attitudes and purchase behavior of organic products.*

### 2.8. Research Model

**Figure 1:**
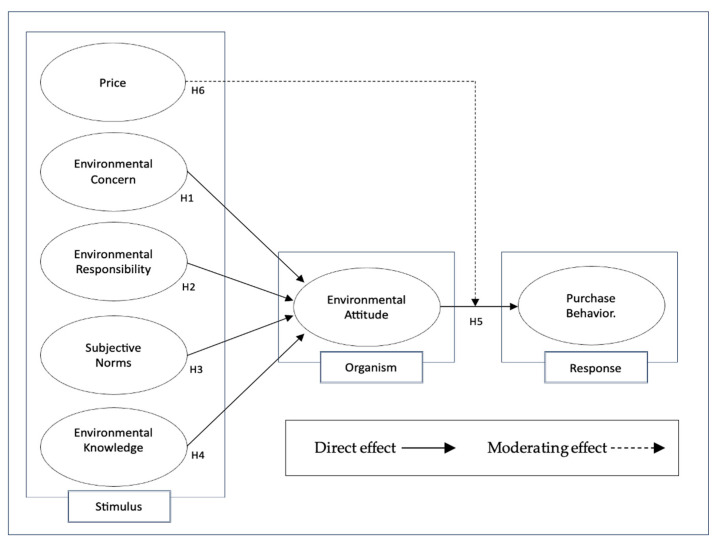
Research hypothesis model.

The present study makes a novel contribution by integrating the SOR model to evaluate how different factors (EC, ER, SN, and EK) act as stimuli that shape EA, which in turn influences the PBOP of organic products. Unlike previous studies conducted primarily in developed economies, this study focuses on Ecuadorian consumers, thereby providing evidence within an emerging market context in which PR is consolidated as a critical factor. In this regard, the inclusion of PR as a moderating variable represents a distinctive contribution, offering a less-explored perspective on the mechanisms that either hinder or strengthen the translation of EA into sustainable purchasing decisions. Figure 1 shows the proposed model.

## 3. Methodology

### 3.1. Instrument Design and Data Collection

To verify the validity of the hypothetical model empirically, a quantitative correlational study with a cross-sectional design was conducted using a 25-item questionnaire adapted to Spanish. This questionnaire was administered to consumers who were identified as buyers of organic products. Prior to data collection, the survey was validated by two experts (one in marketing and one in Research Methodology), and no objections were raised. After obtaining expert approval, a pilot test was conducted with 25 participants to confirm the clarity of the questions.

The study sample consisted of 329 valid responses obtained from 356 questionnaires distributed to consumers of Ecuadorian organic products of the four most populated cities in Ecuador, representing an effectiveness rate of 92.4%. Data were collected through a self-administered online survey, and responses were carefully filtered to exclude incomplete or inconsistent entries. The participants were selected through non-probability convenience sampling to ensure accessibility and feasibility. It is acknowledged that the use of non-probability convenience sampling of university students limits external validity and the generalization of findings to the entire Ecuadorian population. Nevertheless, its theoretical relevance is justified for two reasons: (a) in Ecuador, the adoption of organic products is concentrated among young urban consumers with higher education and relative income, who act as early adopters and represent the core market of the modern retail channel; and (b) to test the SOR mechanism and the moderating effect of PR on the attitude-purchase gap, it is methodologically appropriate to focus on a theoretical case in which sufficient variance in attitudes and exposure to organic product offerings exists. The study population comprised undergraduate and postgraduate students who reported consuming organic products. The inclusion criteria were as follows: participants over 18 years of age who had purchased organic products within the previous month. Data were collected using Google Forms. Assessment and validation of the structural model were performed using SmartPLS 4.

### 3.2. Measures

The items for each variable were adapted from measurement scales used in previous studies. A five-point Likert scale was employed, ranging from 1 = “strongly disagree” to 5 = “strongly agree.” Four items were used to measure EC, four for ER, and four for SN, adapted from Ogiemwonyi et al. [17]. EK was measured using four items from Simanjuntak et al. [104]. PR was assessed using four items adapted from Marde and Verité [105]. EA was measured using four items, and PBOP was measured using a single item, both adapted from Carrión and Arias [3]. See Appendix A.

### 3.3. Statistical Procedure

To estimate and evaluate the hypothetical model, this study employed the Partial Least Squares Structural Equation Modeling (PLS-SEM) method [106]. We developed a hypothetical model using this statistical technique. Model evaluation was performed following the methodological procedures and critical values proposed by Hair et al. [107]. Unlike covariance-based SEM (CB-SEM), PLS-SEM does not require strict assumptions about the residual distribution, allows the use of formative or reflective measurement scales, and is more suitable for moderate sample sizes. Furthermore, it is considered appropriate for analyzing complex models that integrate theoretical relationships with empirical data. In accordance with the methodological recommendations of Leguina [108], a two-stage approach was adopted. In the first stage, the measurement model was assessed to verify its internal consistency, convergent validity, and discriminant validity. In the second stage, the structural model is evaluated to test the proposed hypotheses using PLS-SEM.

To evaluate potential Common Method Bias (CMB), the study performed Harman’s single-factor test via exploratory factor analysis, entering all indicators into an unrotated principal-components solution. The first factor explained 32.7% of the total variance, well below the commonly cited 40% threshold for serious CMB concerns [109], indicating that common method variance is unlikely to threaten the validity of the results. Additionally, the study applied procedural remedies during survey design to mitigate bias, including guaranteeing respondent anonymity, randomizing item order, and adapting items from previously validated scales.

## 4. Results

### 4.1. Demographic Characteristics of the Participants

The sample comprised 329 Ecuadorian consumers. Regarding geographic distribution, priority was given to the participants residing in the country’s four most populous cities. The largest proportion of respondents came from Santo Domingo de los Colorados (32.8%), followed by Quito (25.2%), Guayaquil (18.5%), and Cuenca (4.3%), while 19.1% reported living in other cities across the country. In terms of educational level, 71.7% of the respondents had completed undergraduate studies, while 28.3% had attained a postgraduate degree, indicating a participant profile with a high level of academic training.

With respect to age, 44.7% of the participants were born after 2000 (centennials), followed by 22.5% born between 1979 and 1988 (older millennials), 13.7% between 1995 and 2000 (younger millennials), 8.5% between 1989 and 1994 (mid-millennials), and 10.6% in 1978 or earlier (Generation X). This reflects a sample composed primarily of millennials and centennials, which are considered key segments in the consumption of organic products. In terms of gender, the distribution was balanced: 49.8% identified as male, 49.5% as female, and 0.6% reported another gender identity. This balanced composition allowed for analysis without significant gender-related biases in the study variables. Table 1 provides a detailed overview of the demographic results.

### 4.2. Estimation of the Measurement Model

Statistical tests such as Cronbach’s Alpha (CA), Composite Reliability (CR), and Average Variance Extracted (AVE) were employed to assess the reliability and convergent validity of the measurement model. The values obtained for Cronbach’s alpha and CR (Rho_a and Rho_c) exceeded the threshold of 0.70, thus meeting the standards established in the literature [107]. Similarly, all standardized factor loadings of the indicators were above 0.70, providing strong evidence of satisfactory reliability for the variables included in the study. Also, the study, checked for multicollinearity among constructs in the structural model using full collinearity VIFs and internal relationship VIFs, adopting the conservative threshold of VIF < 3.3. All predictors were below 3.3 [110]. Convergent validity was ensured by confirming that AVE values were greater than 0.50, the minimum acceptable level proposed by Hair et al. [107]. Thus, the results reflected adequate levels of internal consistency and convergent validity. Furthermore, recent research has demonstrated that when AVE values are greater than 0.50, and lower than those of CR, convergent validity is more robustly confirmed [111] (See Table 2).

Discriminant validity was verified using the criterion proposed by Fornell and Larcker, which states that the square root of AVE should exceed the correlation values between each pair of constructs in the model. As shown in Table 3, all square root values on the main diagonal were greater than the correlations with the other constructs, thereby confirming discriminant validity. Additionally, the HTMT index (widely recommended in the literature) was applied, which established a maximum acceptable value of 0.90 [111]. According to the results presented in Table 3, all HTMT values remained below this threshold, further corroborating the discriminant validity of the variables included in the model (See Table 3).

### 4.3. Structural Equation Modeling (SEM): Model Fit and Hypothesis Testing

After analyzing the psychometric properties of the instrument, a structural model was developed. For this purpose, SmartPLS software 4 [112] was employed, applying a bootstrapping resampling procedure with 5000 samples, which allowed the assessment of the statistical significance of the hypothesized causal relationships. The software was also used to calculate the variance in the dependent variables explained by the independent variables and other relevant constructs [110].

With regard to the predictive capacity of the structural model, the R^2^ values were examined following the guidelines of Falk and Miller [113], who established that values must exceed the threshold of 0.10, which is considered acceptable, because lower values, even if statistically significant, do not meet quality standards. In addition, the Standardized Root Mean Square Residual (SRMR) index was calculated, which is recognized as a goodness-of-fit measure in PLS-SEM that helps prevent model misspecifications [114]. According to the literature, an SRMR value below 0.08 indicates a good model fit [107].

The results derived from the analysis of the relationships among the six variables in the hypothesized model supported the acceptance of all the proposed hypotheses. Specifically, PLS-SEM estimates indicated that EC (β = 0.140, *p* < 0.05), ER (β = 0.149, *p* < 0.05), SN (β = 0.206, *p* < 0.05), and EK (β = 0.490, *p* < 0.05) significantly influenced EA. Furthermore, it was confirmed that EA (β = 0.491, *p* < 0.05) influences PBOP and that PR (β = 0.099, *p* < 0.05) moderates the relationship between EA and PBOP. By the other hand, the SRMR value of 0.076 indicates a good model fit, reflecting a low level of discrepancy between the observed correlations and those expected by the theoretical model. Finally, all R^2^ values exceeded the 0.10 threshold, confirming the predictive power of the endogenous variables. Additionally, the study incorporates effect size (f^2^) and predictive relevance (Q^2^) to strengthen the model validity report. The results show that the model’s key relationships exhibit medium (>0.02 to <0.15) and high (>0.15 to <0.35) f^2^ values, and that all endogenous constructs achieved Q^2^ > 0, indicating predictive relevance (See Table 4).

**Figure 2:**
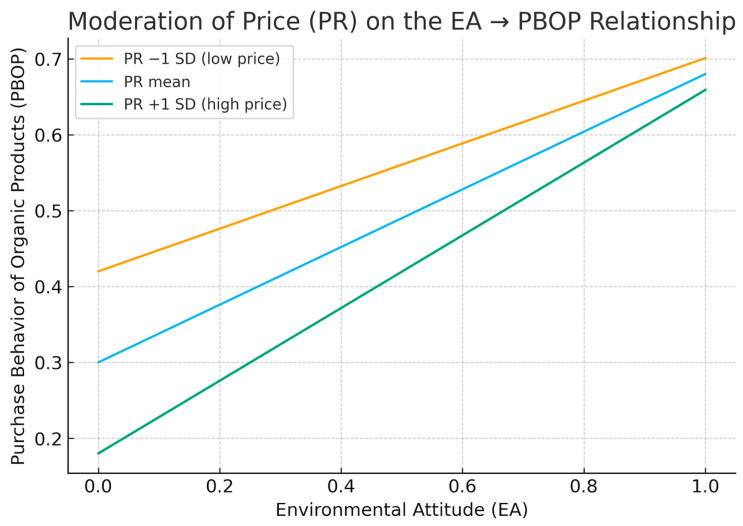
Graphical plot.

By the other hand, the interaction between PR and EA-PBOP was statistically significant yet small (β = 0.099, f^2^ = 0.030, *p* < 0.05). In this sense, simple-slope analyses (Figure 2) indicate that when PR is low, the EA-PBOP slope is steeper, facilitating the translation of favorable attitudes into purchase behavior; conversely, at high PR, the slope flattens, consistent with stronger affordability frictions that widen the attitude–behavior gap.

## 5. Discussion

To facilitate an understanding of the discussion of the findings, the results are presented in response to the following research sub-questions: (a) How do EC, ER, SN, and EK influence consumers’ EA? (b) How does EA influence the PBOP? (c) How does PR moderate the relationship between EA and PBOP?

### 5.1. Influence of Environmental Concern, Environmental Responsibility, Subjective Norms, and Environmental Knowledge on Consumers’ Environmental Attitude

Based on the statistical analyses conducted, this study supports hypothesis H1, indicating EC significantly influences EA among consumers. This finding demonstrates that EC has become a central cognitive antecedent integrating both public and private awareness of environmental issues, while also motivating the adoption of favorable evaluations toward organic consumption. From the theoretical perspective of the SOR, EC functions as a stimulus capable of shaping the organism’s internal state, thereby guiding consumers to make decisions aligned with responsible consumption. Consequently, its relevance is amplified within a sample predominantly composed of millennials and centennials, cohorts widely recognized in the literature as more inclined toward sustainable alternatives, particularly when their EA is strong and consistent. Considering these aspects, this result consolidates EC as the attitudinal foundation that sustains preferences for organic products in emerging market contexts and, at the same time, aligns with the evidence synthesized in the literature review, confirming that EC is positively associated with both the intention to purchase organic products and the adoption of responsible consumption practices. In this regard, the evidence supports the notion that environmental and social values intensify the translation of EC into pro-environmental behaviors, while perceptions of ecological risk foster transformations in consumers’ beliefs and actions. Taken together, this finding provides relevant empirical evidence on consumer behavior in an emerging economy and reinforces the academic literature that has consistently identified EC as a strong determinant of EA [20,37,40,42,44,45].

Similarly, the study supported hypothesis H2, indicating that ER influences EA. This result establishes ER as a sense of duty and commitment to environmental protection, expressed through the willingness to adopt responsible behaviors and preserve natural resources. From the theoretical perspective of the SOR framework, the study reveals that ER functions as an internalized stimulus that transforms ecological concern into a favorable attitudinal predisposition, demonstrating that ER has become a key determinant in the formation of pro-environmental attitudes. Furthermore, the relevance of this finding is reinforced by the profile of the sample included in the study, since consumers belonging to generational cohorts such as millennials and centennials have emerged as segments that tend to exhibit a stronger inclination to purchase organic products, driven by a relatively greater willingness to actively address environmental issues. In light of these considerations, ER provides moral coherence to consumers’ EA, strengthening the evaluative judgments that support their preferences for organic products and, consequently, enhancing their orientation toward more sustainable consumption decisions. The present finding reinforces previous research indicating that ER is associated with pro-environmental behaviors and with the consolidation of a “social environmentalism” ethos that integrates both individual and collective responsibilities, confirming that ER has become a key factor that significantly influences EA [15,49,50,51,52,53,54,55,56,58,59,60].

Continuing the discussion, the study supports hypothesis H3, confirming that SN exert a significant influence on EA among consumers. This result is both conceptually and empirically relevant, as it deepens the understanding of how social mechanisms operate as powerful drivers of attitudinal change toward ER. From the theoretical perspective of the SOR framework, the study demonstrates that SN function as external stimuli that activate internal cognitive and affective processes, translating social expectations into pro-environmental orientations. In this sense, the research expands upon previous theoretical insights by emphasizing that social pressure not only reinforces EC but also confers normative legitimacy on sustainable consumption choices. The findings of this study reveal that within a generational context dominated by millennials and centennials, the influence of SN gains greater interpretative weight, as these cohorts tend to construct their consumption identities in response to perceived social expectations. This dynamic reflects the coexistence of social validation and environmental awareness as mutually reinforcing forces. Therefore, the influence of SN on EA observed in this study can be interpreted as a manifestation of collective moral signaling, where sustainability emerges as a social marker of identity and belonging rather than a mere ethical preference. However, the SN-EA relationship should not be viewed as universally stable, as the literature presents contrasting evidence that questions the predictive strength of SN across different contexts. Although several studies have documented their positive and significant impact on EA and GPOP, highlighting the role of normative influence in shaping sustainable choices [6,42,63,64,65,66,68]. While, other authors have reported weak or nonsignificant associations, arguing that individual moral conviction and EK may overshadow social pressure as behavioral determinants [69,70,71,72]. These inconsistencies suggest that the explanatory power of SN may depend on sociocultural context, levels of environmental awareness, and the maturity of the organic market.

The statistical analyses also supported hypothesis H4, confirming that EK exerts a significant positive influence on EA. This finding contributes to the ongoing theoretical debate by reaffirming that cognitive awareness represents a central determinant in the attitudinal formation process toward sustainability. Within the SOR framework, the study demonstrates that EK operates as a crucial cognitive stimulus that enhances consumers’ ability to interpret the environmental consequences of their choices, thereby translating knowledge into evaluative and affective dispositions consistent with responsible consumption. This theoretical mechanism underscores that understanding precedes motivation, and that informed consumers are more likely to internalize ecological values as guiding principles of their behavior. In this regard, the empirical relevance of the EK-EA relationship is accentuated by the characteristics of the sample, composed primarily of millennials and centennials, consumers with greater access to environmental information, which fosters the integration of ecological reasoning into their consumption identity. Therefore, the study advances the debate by illustrating that EK not only informs but also strengthens EA among consumers in emerging economies. This finding demonstrates that EK emerges not only as a cognitive variable but also as a moral and cultural facilitator of sustainable consumption, bridging knowledge and attitudinal commitment in contexts where formal environmental education is still developing. However, this result should be critically examined in light of the heterogeneity observed in previous research. The findings of this study align with prior evidence showing that consumers with higher EK display greater sensitivity to green marketing initiatives and heightened discernment toward environmentally identified products [24,35,79,81,83,86,87]. Conversely, they contrast with other studies that question the universality of this linkage, in which some authors argue that EK alone does not necessarily translate into behavioral change, as emotional engagement, situational constraints, and perceived efficacy often moderate this relationship [69,71,88,89,90].

### 5.2. Influence of Environmental Attitude on the Purchase Behavior of Organic Products

The statistical analyses supported hypothesis H5, confirming that EA exerts a significant positive influence on PBOP. This result holds strong theoretical implications within the SOR framework, as it empirically validates the mechanism through which internal attitudinal states (organism) transform environmental into observable behavioral responses. In this sense, EA functions as a psychological bridge that translates cognitive and affective evaluations of sustainability into tangible purchasing behaviors. This interpretation advances the scientific understanding of the SOR model by emphasizing that the organismic component is not merely reactive but actively mediates and internalizes environmental stimuli, shaping consistent pro-environmental actions. From a generational and contextual standpoint, this effect becomes particularly salient among millennials and centennials, who constitute the majority of the sample. These cohorts, characterized by high levels of education and digital exposure to sustainability discourse, tend to express their EA behaviorally through consumption patterns that reflect moral identity and social signaling. Thus, the finding contributes to the refinement of attitudinal models of sustainable behavior by evidencing that EA serves as a stable internal driver capable of overcoming structural or perceptual barriers commonly found in emerging markets, such as Ecuador. In contexts where sustainable consumption is often constrained by affordability concerns, information asymmetries, and limited organic product availability, the internalization of ecological attitudes becomes a decisive mechanism for behavioral activation. In this sense, this study contributes to the ongoing theoretical dialog by positioning EA as the organismic core of the SOR process, capable of mediating and amplifying the influence of prior cognitive variables on behavioral outcomes. This strengthens the conceptual argument that behavioral sustainability cannot be fully explained by external stimuli alone, but rather by the degree to which consumers integrate ecological values into their self-concept and decision-making schema. Therefore, the present finding not only reinforces the psychological validity of the SOR paradigm but also provides empirical evidence of its contextual adaptability in emerging economies, where structural limitations coexist with rising environmental awareness. Overall, these results align with prior empirical evidence that underscores the positive association between EA and PBOP, confirming the pivotal role of EA in mediating the path from ecological cognition to responsible consumption behavior [6,9,12,17,24,37,48,71,90,91,92,93].

### 5.3. Moderating Effect of Price on the Relationship Between Environmental Attitude and the Purchase Behavior of Organic Products

The statistical analyses supported Hypothesis H6, confirming that PR moderates the relationship between EA and PBOP. This result carries notable theoretical implications, as it substantiates one of the core assumptions of the SOR model: that external stimuli can amplify or attenuate the behavioral outcomes derived from internal psychological states. Within this theoretical lens, PR operates as a contextual stimulus that interferes with the attitudinal pathway between organismic responses (EA) and behavioral expressions (PBOP), revealing the fragility of pro-environmental intentions when confronted with economic constraints. Hence, even when consumers internalize ecological values and exhibit favorable attitudes toward sustainable consumption, their behavioral translation is not immune to structural and financial pressures. From a critical standpoint, this finding contributes to refining the SOR model by demonstrating that not all stimuli are positively reinforcing; rather than sensitivity PR can exert an inhibiting effect on behavioral activation. In doing so, the study expands the understanding of the dual nature of stimuli in consumer-environmental psychology, showing that economic stimuli act as *boundary conditions* that determine whether EA are transformed into actual purchasing behavior. This insight strengthens the argument that the relationship between cognition, attitude, and behavior is neither linear nor unidirectional, but instead dynamically contingent on situational variables such as perceived affordability and value assessment. By the other hand, this study enriches the SOR framework by positioning PR not merely as a transactional variable, but as a contextual moderator that shapes the translation of psychological predispositions into action. It thereby advances the theoretical understanding of how external market stimuli interact with internal motivational states, offering a nuanced interpretation of the barriers that mediate sustainable consumer behavior. In doing so, the findings provide empirically grounded evidence that contributes to a more context-sensitive comprehension of the mechanisms underlying organic consumption. Consistent with prior empirical research, the results corroborate that PR remains a critical determinant of sustainable purchasing decisions, as consumers’ willingness to act pro-environmentally often fluctuates according to their perceived economic feasibility and value-for-money evaluations [3,6,7,12,15,19,26,27,67,98,101,102,103].

## 6. Conclusions

This study deepens our understanding of purchasing behavior for products aligned with environmental protection in emerging markets such as Ecuador. It critically demonstrates how EA interact with cognitive, affective, and contextual stimuli to shape PBOP. Drawing on the SOR model, the research integrates multiple antecedents (EC, ER, SN, and EK) to explain how individual cognition and social influence converge in the formation of EA. This multidimensional perspective expands upon previous models by empirically validating that EA arise from a complex interplay of awareness, responsibility, and normative orientation, rather than from isolated motivational factors.

Furthermore, the study identified PR as a decisive moderating stimulus that reveals the economic limits of attitudinal translation. The finding that favorable EA do not always manifest themselves in purchasing actions when sustainable products are perceived as expensive offers nuanced theoretical and practical insights into the intention-behavior gap, a persistent, underexplored challenge in the context of emerging Latin American economies. By demonstrating that economic stimuli can attenuate the behavioral effects of internal attitudes, this study contributes to refining the SOR model, showing that external market forces can neutralize pro-environmental predispositions even among young and educated consumers.

The results therefore elevate the field by situating the psychological mechanisms of sustainable behavior within a developing economy, providing one of the few empirical validations of the SOR model in a Latin American context. This not only enriches theoretical generalization but also introduces cultural and economic nuances often neglected in Western-focused research. Consequently, the study bridges a critical gap between environmental psychology and consumer behavior in emerging markets, offering an integrative perspective that links ecological consciousness, social influence, and structural constraints in shaping responsible consumption.

Finally, beyond its theoretical contribution, this work underscores the urgency of strategic interventions, both managerial and policy, that align affordability with sustainability, ensuring that pro-environmental values are translated into tangible market behaviors. By contextualizing the SOR framework in Ecuador’s evolving sustainability landscape, this study goes beyond mere replication to offer a contextualized contribution that broadens the global dialog on how ecological values translate into action in diverse economic realities.

### 6.1. Practical, Theoretical, and Social Implications

Theoretically, this study advances the understanding of sustainable consumption by extending the SOR model to explain how cognitive (EC, ER, EK) and social stimuli (SN) activate attitudinal mechanisms that foster EA and its translation into PBOP. A key theoretical contribution lies in clarifying the role of PR within this framework: rather than functioning solely as an external stimulus, PR operates as a situational moderator that conditions the strength and direction of the response. In pro-ecological contexts, PR acts as a boundary factor that determines whether positive attitudes are converted into behavioral outcomes, revealing that some environmental cues can attenuate rather than stimulate pro-environmental action. This reinterpretation deepens the SOR model by incorporating situational constraints as integral components of behavioral formation, demonstrating that sustainable behavior emerges from the dynamic interplay between internal motivations and external conditions. Consequently, this study refines the theoretical applicability of the SOR framework beyond developed economies, offering a more context-sensitive explanation of the attitude–behavior gap in green consumption within emerging markets.

At the managerial and business levels, the findings offer concrete strategic guidance for closing the attitude–behavior gap in the organic product market. The evidence that positive EA can be weakened by PR suggests that firms must adopt value-based pricing strategies that highlight the long-term functional and social benefits of organic products rather than competing solely on cost. Managers should develop tiered pricing structures and smaller package sizes to make organic options more accessible to price-sensitive segments, particularly in emerging markets. Additionally, firms can implement cost-optimization innovations in local sourcing, logistics, and production to reduce PR differentials without compromising product integrity. From a communication perspective, marketing strategies should emphasize total value (including health, environmental, and ethical dimensions), thereby reframing organic consumption as both a personal and collective investment. Targeted campaigns can leverage the predisposition of millennials and centennials toward social and ER by employing cause-related marketing, peer endorsement, and digital storytelling to reinforce symbolic and emotional value while diminishing perceptions of economic sacrifice.

From a social and policy standpoint, the results underscore that fostering favorable EA is insufficient without structural mechanisms that facilitate PBOP. Policymakers should align interventions with national sustainability and circular economy strategies, promoting tax incentives, subsidies, and certification support for organic producers to improve affordability and supply chain competitiveness. In parallel, consumer education initiatives (integrated into national environmental curricula and community awareness programs) can strengthen environmental literacy and reshape consumption norms toward sustainability. The empirical evidence from Ecuador highlights the need for public–private collaboration to democratize access to sustainable products, ensuring that organic consumption is not perceived as a privilege but as a viable lifestyle choice across socioeconomic strata.

### 6.2. Limitations and Future Research

Although the results of this study provide significant evidence regarding the consumption of organic products in the Ecuadorian context, several limitations of this study should be acknowledged. First, the methodological design was cross-sectional and based on self-reported data, which restricts the ability to establish strong causal relationships, and may be subject to social desirability bias. Second, the study relied on a non-probabilistic sample composed primarily of millennials and centennials with academic training, which limits the generalizability of the findings to other population segments with different sociodemographic characteristics. Finally, the study focused exclusively on PR as a moderating variable, excluding other contextual factors (such as product availability, certifications, or the influence of marketing campaigns) that could also condition the relationship between EA and purchasing behavior.

Building on these limitations, future research should consider the use of longitudinal or experimental designs to analyze the evolution of consumer attitudes and behaviors over time or to assess the causal effects of specific interventions. It is also relevant to diversify samples by including consumers from different socioeconomic levels, regions, and age groups to gain a broader and more representative understanding of the market. Additionally, incorporating other moderating or mediating variables (such as trust in certifications, perceptions of quality, influence of public policy, or exposure to environmental education campaigns) would enrich the understanding of the phenomenon. Finally, extending the application of the model to other emerging markets in Latin America would allow for comparisons across different sociocultural contexts and would provide more robust evidence to support the design of global strategies aimed at fostering sustainable consumption.

## Figures and Tables

**Table 1:** Demographic data.

Characteristics	Category	#	%
City	Santo Domingo de los Colorados	108	32.8%
	Quito	83	25.2%
	Guayaquil	61	18.5%
	Cuenca	14	4.3%
	Other cities in Ecuador	63	19.1%
Educational level	Under graduate	236	71.7%
	Postgraduate	93	28.3%
Age range	1978 or earlier	35	10.6%
	Between 1979 and 1988	74	22.5%
	Between 1989 and 1994	28	8.5%
	Between 1995 and 2000	45	13.7%
	After 2000	147	44.7%
Gender	Male	164	49.8%
	Female	163	49.5%
	Other	2	0.6%
n =	329		

**Table 2:** Convergent validity and reliability.

Variable	Item	Outer Loading	T Statistics	CA	VIF	Rho_a	Rho_c	AVE
EC	EC1	0.774	17.901	0.726	2.224	0.781	0.832	0.623
	EC2	0.776	16.766		2.249			
	EC3	0.817	29.055		1.149			
ER	ER1	0.806	34.355	0.807	1.770	0.814	0.874	0.634
	ER2	0.748	23.701		1.477			
	ER3	0.760	18.211		1.600			
	ER4	0.866	55.729		2.185			
SN	SN2	0.889	57.882	0.844	2.196	0.845	0.906	0.763
	SN3	0.866	44.647		1.957			
	SN4	0.865	41.364		1961			
EK	EK1	0.845	39.351	0.812	1.780	0.826	0.888	0.726
	EK2	0.817	25.102		1.666			
	EK3	0.893	73.512		2.008			
EA	EA1	0.891	60.600	0.858	2.396	0.868	0.902	0.778
	EA2	0.902	61.441		2.542			
	EA3	0.853	43.594		1.845			
PR	PR1	0.842	40.218	0.888	2.067	0.890	0.923	0.749
	PR2	0.887	54.162		2.707			
	PR3	0.887	43.812		2.803			
	PR4	0.846	40.660		2.195			
PBOP	US1	1.000	-	1	1	1	1	1

**Table 3:** Discriminant validity.

	EC	ER	SN	EK	EA	PR	PBOP
**EC**	0.789	0.678	0.404	0.828	0.712	0.479	0.503
**ER**	0.544	0.796	0.722	0.707	0.749	0.527	0.552
**SN**	0.345	0.598	0.873	0.448	0.635	0.625	0.576
**EK**	0.666	0.568	0.403	0.852	0.894	0.679	0.601
**EA**	0.619	0.627	0.541	0.751	0.882	0.730	0.693
**PR**	0.445	0.446	0.541	0.579	0.638	0.865	0.605
**PBOP**	0.460	0.449	0.549	0.542	0.641	0.571	1.000

Note: Fornell and Larcker on the diagonal; HTMT values above the diagonal.

**Table 4:** Results of hypotheses testing.

Hypotheses	Relation	β	f^2^	*p*-Values	Hypotheses
H1	EC-EA	0.140	0.030	0.002	Accepted
H2	ER-EA	0.149	0.031	0.009	Accepted
H3	SN-EA	0.206	0.079	0.000	Accepted
H4	EK-EA	0.490	0.348	0.000	Accepted
H5	EA-PBOP	0.491	0.265	0.000	Accepted
H6	PR (EA-PBOP)	0.099	0.030	0.000	Accepted

SRMR: 0.076; R^2^(EA): 0.655; R^2^(PBOP): 0.446; Q^2^(EA): 0.194; Q^2^(PBOP): 0.067.

## Data Availability

The original data presented in the study are openly available in https://drive.google.com/drive/folders/1-7jsJpOHXr77y64wHPofOZ1WLz3G_yEf?usp=drive_link (accessed on 19 September 2025).

## Appendix A

**Table A1:** Survey questions.

EC [17]	EC1	I am particularly concerned about the natural environment.
	EC2	Human beings are seriously misusing the natural environment.
	EC3	I will reduce my consumption to help protect the environment.
	EC4	A major action is required to safeguard the environment.
ER [17]	ER1	Supporting environmental protection makes me feel like being environmentally responsible.
	ER2	I should be responsible for protecting our environment.
	ER3	Environmental protection starts with me.
	ER4	I am passionately involved in environmental protection issues.
SN [17]	SN1	I would say that buying eco-friendly products will benefit me and other people.
	SN2	People whose opinions matter would prefer that I buy eco-friendly products.
	SN3	I would say that buying eco-friendly products is compulsory.
	SN4	My friend strongly determines whether I should buy a green product or not.
EK [104]	EK1	Know the environmental issues
	EK2	Know how to choose products that can reduce the waste
	EK3	Understood the symbols or signs of green products on product packaging
	EK4	Know that green products cause less damage than other products
PR [105]	PR1	I am not willing to pay more for organic products.
	PR2	I cannot afford to pay more for organic products.
	PR3	Organic products are still too expensive.
	PR4	For me, the price of organic products is often a problem.
EA [3]	EA1	I think organic products help save nature and its resources.
	EA2	Environmental protection is important to me when shopping for products.
	EA3	I have a favorable attitude toward purchasing organic products.
	EA4	If I get a choice, I’ll prefer an organic product over a conventional product.
PBOP [3]	PBOP1	I’ve bought organic products regularly.
